# Effects of Sulfamethoxazole on the Microbial Community Dynamics During the Anaerobic Digestion Process

**DOI:** 10.3389/fmicb.2020.537783

**Published:** 2020-09-16

**Authors:** Valentina Mazzurco Miritana, Giulia Massini, Andrea Visca, Paola Grenni, Luisa Patrolecco, Francesca Spataro, Jasmin Rauseo, Gian Luigi Garbini, Antonella Signorini, Silvia Rosa, Anna Barra Caracciolo

**Affiliations:** ^1^Water Research Institute, National Research Council, Montelibretti, Italy; ^2^Department of Energy Technologies, Italian National Agency for New Technologies, Energy and Sustainable Economic Development, Rome, Italy; ^3^Institute of Polar Sciences, National Research Council, Montelibretti, Italy

**Keywords:** cattle manure, sulfamethoxazole, biogas, anaerobic digestion, microbial community, antibiotic degradation, ARGs

## Abstract

Anaerobic digestion (AD) treatment of cattle manure and slurry makes it possible to produce biogas, a renewable and storable biofuel, as well as digestate, a residual organic matter that can be used to replace chemical fertilizers. On the other hand, the intense use of antibiotics (e.g., sulfamethoxazole) in animal husbandry practices is showing increasing negative impacts resulting from the release of still metabolically active molecules into agroecosystems. In the present study, cattle manure collected from an AD plant-feeding tank was used as feedstock for AD experiments in which some batches were spiked with 5 mg L^–1^ of sulfamethoxazole (SMX). Adding the antibiotic affected the microbial community dynamic; in particular, the efficiency of the acidogenic and acetogenic phases of the process corresponded to higher CH_4_ and H_2_ production than in the control. SMX was also degraded, and at the end of the experiment (69 days), just 20% of its initial concentration was found. The relative abundance (ARG/16S) of resistance genes *sul1*, *sul2*, and the proxy *intI1* initially found in the ingestate decreased during the AD in both the spiked and control batches, suggesting that this process lowers the likelihood of antibiotic resistance genes spreading.

## Introduction

Anaerobic digestion (AD) is an efficient process that transforms organic waste, e.g., agriculture and breeding residues, into renewable energy (biogas) and digestate (Guo et al., 2015; Sui et al., 2017; European Biogas Association, 2018; Styles et al., 2018). The latter, containing considerable amounts of plant-essential macro- and micro-nutrients, is a good alternative to chemical fertilizers (Bustamante et al., 2012; Barra Caracciolo et al., 2015; Bharathiraja et al., 2018).

Other advantages of AD are the significant reduction of pathogens in the digestate, a complete recycling of organic waste, and mitigation of global CO_2_ emissions, in line with the circular economy approach (Pampillón-González et al., 2017). In Italy, digestate is commonly used as a fertilizer, and its application in agriculture is regulated by the Inter-Ministerial Decree No. 5046/2016, which lists some parameters (e.g., total nitrogen and phosphorous, Pb, Cd, Ni, Zn, Cu, Hg, and Cr) to be checked. Recently, the EU Fertilizing Products Regulation (EU 2019/1009) has been proposed for promoting digestate use over all Europe. However, the possible presence of emerging contaminants, such as antibiotic residues, has not been considered so far. In fact, the environmentally friendly application of digestate on agricultural soil does not exclude the likelihood of introducing antibiotics, such as sulfamethoxazole (SMX); the latter is commonly used in livestock production worldwide (Rauseo et al., 2019).

Antibiotic residues are found in animal waste (Widyasari-Mehta et al., 2016), and consequently, antibiotic-resistant bacteria and antibiotic resistance genes (ARGs) can enter farm digesters, where their effect and fate are not well known. The AD process relies on a complex microbial community, in which different functional groups cooperate in sequential metabolic steps with a final production of methane-rich biogas (Madigan et al., 2014). Antibiotics are reported to reduce microbial activities or influence organic matter degradation during biogas production (Ji et al., 2013; Grenni et al., 2018). Among the functional microbial groups acting in AD, methanogens, which have the slowest growth rates, are the most sensitive to any changes in process conditions (Bharathiraja et al., 2018). They control the last and critical step of AD, and the proportion of *Bacteria*/*Archaea* is related to the process efficiency (Ferraro et al., 2019). Some research evaluated the effect of SMX on AD performance: a concentration of 500 mg L^–1^ produced a complete inhibition of methane production (Cetecioglu et al., 2012), and lower amounts (45–50 mg L^–1^) caused high accumulation of volatile fatty acids (VFAs) and a consequent lowering of the pH, thus altering the AD process efficiency (Aydin et al., 2015). Lower antibiotic concentrations (ranging from 1 to 10 mg L^–1^) only partially impacted the microbial community and AD process (Cetecioglu et al., 2012).

Other research demonstrates that SMX can be degraded during AD, depending on the specific experimental conditions and its initial concentration (Mohring et al., 2009; Cetecioglu et al., 2015; Cheng et al., 2018). Feng et al. (2017) report that antibiotic residues (e.g., SMX 1 μg L^–1^) were effectively removed during the AD of sludge and manure.

The selection of sulfonamide-resistant bacteria and spread of ARGs (Sköld, 2000; Enne et al., 2002) (*sul1, sul2, sul3*) have been demonstrated in various research on effluents, reclaimed water, and manure (Baquero et al., 2008; Martinez, 2009; Mckinney et al., 2018; Rauseo et al., 2019). However, there has been little published research on digestate, and the results have been quite different (Tasho and Cho, 2016; Grenni et al., 2018). Some authors find that most ARGs are diminished during the AD process except *sul2* (Polesel et al., 2016; Zhang et al., 2018). On the other hand, other authors report that mesophilic AD was able to remove ARGs, including *sul1* and *sul*2, while other ARGs increased (Ma et al., 2011). In this work, the possible effects of SMX on the dynamics of the AD process and H_2_ and CH_4_ production were investigated in batch mode by testing manure samples collected from an AD plant-feeding tank and adding the antibiotic SMX at a final concentration of 5 mg L^–1^. For this purpose, cattle manure was used testing the AD process as both the substrate and inoculum in the AD experiment. Changes in the microbial community structure [fluorescence *in situ* hybridization (FISH) and next-generation sequence (NGS) techniques]; occurrence of *sul1*, *sul2*, and *intI1* genes; and antibiotic degradation were also investigated.

## Materials and Methods

### Ingestate Sampling and Characterization

The organic input for the AD plants consists of cattle manure partially digested in the herbivorous ruminal ecosystem, and for this, it can be named “ingestate.”

The ingestate was collected from the feed pipe of a full-scale continuous stirred-tank reactor plant located on a beef and dairy cattle farm (Lazio, Italy). It was mainly composed of cow manure with a minor fraction (8–10%) of food waste (manager’s communication). The ingestate was mixed, and glass bottles of 2 L each (three replicates) were filled and transported to the laboratory where the batch experiment was immediately set-up. At the same time, some ingestate aliquots (three replicates) were used for its characterization in terms of total and volatile solids (g L^–1^), pH, organic carbon, total nitrogen, water content (%), and microbiological abundance (N. cells mL^–1^). These data are reported in Table 1. Moreover, the initial SMX concentration was determined.

**TABLE 1:** Characterization of the ingestate used for the AD experiment.

Total solids (g L^–1^)	119.0 ± 0.7
Volatile solids (g L^–1^)	104.3 ± 0.7
pH	6.4 ± 0.1
Organic carbon (%)	40.0 ± 0.15
Total nitrogen (%)	3.2 ± 0.3
Water content (%)	88.7 ± 0.08
Metals	Supplementary Table 1
Total microbial abundance (N. cells mL^–1^)	1.89E+09 ± 3.0E+08

### AD Experiment

The experiment was set-up in batch mode using six Pyrex reactors of 600 mL each (three replicates for each experimental condition) for evaluating the AD performance of the ingestate collected from an AD plant-feeding tank and of the latter with SMX. Each reactor was filled with 300 mL of the ingestate (corresponding to 31 g VS) without pretreatment (e.g., no filtration, no dilution) in order to mimic a full-scale biogas plant condition. The antibiotic SMX was added to three batches (SMX batches). For this purpose, we dissolved SMX (SMX purity 99%, Sigma-Aldrich S7507) powder in a methanol/MilliQ solution (stock solution); then, we further diluted the stock solution in ultrapure water. The final amount of methanol in each batch was very low (0.18 in 300 mL of working volume).

Aliquots of the SMX solution were added to each batch in order to reach a final concentration of 5 mg L^–1^. This concentration was used because, in other works (Cetecioglu et al., 2012), it did not completely inhibit the AD, and it was also close to a concentration found in cattle manure in another previous research (Rauseo et al., 2019).

The other three batch replicates were considered controls. All the batches were sealed with rubber stoppers and metal rings and then flushed with N_2_ (10′) to establish an anoxic environment. The experiment was carried out in mesophilic (37–38°C) and orbital shaking (100 rpm) conditions. Biogas and organic acid and microbial abundance analyses were performed over the experimental time (69 days). The sampling frequency of the gas and the liquid medium varied in line with the evolution in the AD process. During the first 6 days, the biogas measurements were performed daily. For the next 2 weeks, the measurements were performed every 3 days and then weekly. The experiment ended when no more CH_4_ had been produced for 2 weeks. At selected times (5, 10, 15, 21, and 69 days), slurry aliquots were sampled for SMX determinations and evaluation of the antibiotic effect on the structure of the microbial community. The latter was characterized using both the fluorescence *in situ* hybridization and next-generation sequencing methods. Moreover, the 16S rRNA gene, the resistance genes *sul1* and *sul2*, and the class 1 integron-integrase gene *intl1* were also quantified by quantitative PCR (qPCR).

### Biogas and Organic Acid Measurements

The total biogas volume was measured using a water displacement system (Martínez-Sibaja et al., 2011). The biogas composition in the headspace of the reactors was analyzed using a gas chromatograph (Focus GC, by Thermo Fisher Scientific, United States) equipped with a thermal conductivity detector (TCD) and a 3-m stainless steel column packed with Hayesep Q (800/100 mesh). Nitrogen gas was used as a carrier at a flow rate of 35 mL min^–1^. The temperature of the column and of the injector was 120°C and that of the TCD was 200°C. The cumulative H_2_ and CH_4_ productions were calculated using the Logan equation (Logan et al., 2002). The metabolic products of fermentation, such as acetic, butyric, propionic, lactic, succinic, and formic acids, were analyzed with a high-performance liquid chromatograph (Thermo Spectra System, United States) equipped with both a UV detector (l1/4 210 nm) and a refractive index detector. The column, a 300 mm × 7.8 mm Rezex ROA-Organic Acid Hþ (8%) (Phenomenex, United States) with a 4 × 30 mm security guard cartridge Carbo-H (Phenomenex, United States), was operated at 75°C, using a solution of 5 mN H_2_SO_4_ as the mobile phase (flow rate, 0.5 mL min^–1^). The liquid samples were diluted 1:10 in H_2_SO_4_ 5 mN and filtered with a 0.22-μm membrane before injection into the HPLC.

### Determination of SMX

Sulfamethoxazole was extracted by pressurized liquid extraction (PLE) using the Thermo Scientific Dionex, United States, ASE^TM^ 150 (accelerated solvent extractor) system as described in Rauseo et al. (2019). Briefly, each sample (2 g) was mixed and homogenized with inert diatomaceous earth (Thermo 062819) to completely fill the PLE cell. The extraction solvent was a mixture of water: methanol (50:50, *v/v*). The final extract (about 20 mL volume) was then diluted with pure water to reduce the methanol content to below 5% and was purified following the solid phase extraction (SPE) procedure as reported in detail in a previous work (Rauseo et al., 2019). SMX was eluted from the SPE cartridges (Waters Oasis hydrophilic–lipophilic balance, HLB cartridges, 6 mL, 1 g) using 2 × 1.5 mL methanol-ethyl acetate (1:1 *v/v*) and 2 × 1.5 mL methanol containing 1% ammonia. The eluates were evaporated under a gentle nitrogen stream at room temperature to reach a final volume of about 50 μL and then reconstituted with the mobile phase used for the chromatographic analysis.

Sulfamethoxazole in the purified extracts was analyzed by using high-performance liquid chromatography (HPLC, column oven mod. LC-100 and micro Pump Series 200, Perkin Elmer, United States) equipped with a 7125 Rheodyne injector valve with a 20-μL loop and interfaced to a triple quadrupole mass spectrometer detector connected with an electrospray ionization detector (MS-MS, mod. API 3000, AB Sciex, Germany). The chromatographic Gemini column (150 × 4.6 mm, 5 μm RP C 18, Phenomenex, France), preceded by a guard column packed with the same stationary phase, was maintained at 25°C. The elution was at a 1.0 mL min^–1^ flow rate. The mobile phase was composed of acidified MeOH (0.1% formic acid–phase A), acidified ultrapure water (0.1% formic acid–phase B), and acetonitrile (ACN–phase C) of an HPLC grade. The elution was in an isocratic mode (phases A, B, C: 33, 33, 34%, respectively). The MS/MS detector was set in a positive ESI mode, and the source temperature was set at 400°C. The ionspray voltage was +5 kV, and the nebulizer and curtain gas were at 14 and 12 units, respectively. Nitrogen was used as both a collision and drying gas. Declustering, focusing, and entrance potential were set at 31.2, 75, and 9 V, respectively. Multiple reaction monitoring detection was based on the precursor (254.2 m/z) and product ion transitions (92.0, 108.1, and 156.1 m/z) and on the comparison of the retention time (2.23 min.).

The calibration curve for the target compound was obtained by analyzing working standard solutions at the following concentrations: (0.1, 0.25, 0.5, 1.0, 2.5, 5.0, and 7.5 mg L^–1^). The linearity was confirmed with an *R*^2^ ≥ 0.98 for the concentration range investigated. Deuterated SMX (SMX–d4, Clearsynth) was used as the internal standard in the calibration procedure and to compensate for possible matrix effects.

In order to check the performance of both the extraction/purification and analytical procedures, SMX recoveries from the ingestate matrix, artificially spiked at different concentrations of SMX (1.0, 5.0, and 7.5 mg L^–1^), were determined in triplicate. For each final concentration, triplicated spiked samples were prepared. SMX detection was performed by subtracting the blank level of the ingestate non-spiked sample. SMX recoveries ranged between 85 and 95%, and the overall repeatability (*n* = 3) of the extraction procedure was satisfactory (relative standard deviation value ranging from 1.5 to 7.8%). The limit of detection (LOD) was calculated in accordance with the IUPAC method (Thompson et al., 2002) and was 1.5 μg L^–1^. The quantification limits were set at three times the LOD.

### Total Microbial Abundance

Total microbial abundance (N. cells mL^–1^) was measured with the epifluorescence direct count method, using DAPI (4′,6-diamidino-2-phenylindole) as a DNA fluorescent intercalant. Slurry samples (1 g for three replicates) were collected and transferred to a test tube containing 9 mL of a fixing solution (130 mM NaCl, 7 mM Na_2_HPO_4_, 3 mM NaH_2_PO_4_, 2% formaldehyde (*v/v*), 0.5% Tween 20 (*v/v*), and 100 mM sodium pyrophosphate) and processed as described in detail in a previous work (Barra Caracciolo et al., 2005a). The microscope used was a Zeiss epifluorescence microscope AXIOSKOP 40 (Carl Zeiss, Germany) equipped with a ZEISS HXP 120v light source and 1000× magnification.

### Microbial Community Composition Using Fluorescence *in situ* Hybridization

Samples were collected from each experimental reactor and fixed with a formalin solution (4% *w/v*, 6 h at 4°C) as reported in Pernthaler et al. (2001). After being rinsed, the pellets were resuspended in an ethanol (96%) and PBS (1×) solution and stored until use (−20°C).

Before the FISH analysis, a cell-extraction procedure was performed to detach and separate cells from inorganic particles as described in a previous work (Barra Caracciolo et al., 2005a).

The FISH analysis was carried out as described in detail in Amann et al. (1995) and Barra Caracciolo et al. (2005b). In particular, each sample (three replicates for each condition) was collected onto a membrane filter (white polycarbonate filters, pore size 0.22 μm, diameter 25 mm, Millipore, United States). Each filter was cut into sections, and at least two of them were hybridized with the same probe. Hybridization buffers (5M NaCl, 1M Tris/HCl, 100 g L^–1^ sodium dodecyl sulfate and a specific amount of formamide) with each specific probe were prepared. Oligonucleotide probes for the *Bacteria* (EUB338 I, II, III) and *Archaea* (ARC915) domains were used (Greuter et al., 2016). Moreover, the LGC354 II, III, SRB385, MB1174 probes were also used for the detection of *Firmicutes, Sulfate-Reducing Bacteria* (SRB) (class *Deltaproteobacteria*) and methanogens (*Methanobacteriales*), respectively. These are key microbial taxa involved in the AD process. All oligonucleotide probes (50 ng μL^–1^), labeled with carboxyfluorescein (FAM) or indocarbocyanine (Cy3) dye at the 5′-end, were purchased from MWG AG Biotech, Germany. The filters were mounted on microscope slides and kept in a hybridization chamber for 90 min at 46°C. The filters were then washed in a buffer at 48°C for 15 min, rinsed with distilled water and air-dried. Finally, the slides were mounted with a few drops of VectaShield (Vector Laboratories, United States) and DAPI in order to estimate the proportion of cells targeted by each specific probe out of the total cells. The slides were viewed using the abovementioned Zeiss epifluorescence microscope. The micrographs were obtained using the AxioVision Release 4.6.3 program. The results are expressed as the percentage of DAPI cells that hybridized with the fluorescent probe (% positive cells vs. DAPI).

### DNA Extraction

Total DNA was extracted from each slurry sample using the DNeasy PowerSoil kit (Qiagen, United States) in accordance with the manufacturer’s recommendations. A non-template sample (DNA-free water) was included as a negative control during the whole workflow. The extraction yield and quality of the DNA were assessed with spectrophotometry (Multiskan Sky Microplate Spectrophotometer, Thermo Fisher Scientific, United States). All extractions were stored at −20°C until use. The extracted DNA was used for both the qPCR and NGS analyses.

### Quantitative PCR of SMX Resistance Genes

Quantitative PCR was used to quantify two SMX-resistance genes (*sul1* and *sul2*), and the class 1 integron-integrase gene (*intl1*). In addition, the 16S rRNA gene copy numbers were determined to assess the overall microbial load and to calculate the relative abundance of the resistance genes targeted in the samples collected. All qPCR assays were performed in the CFX96 real-time PCR detection system (Bio-Rad, United States) using SYBR green detection. The primers used for the ARGs, 16S, and *intl1* gene quantification are listed in Supplementary Table 2. Each reaction was carried out in a total volume of 20 μL containing 10 μL SsoAdvanced Universal SYBR Green Supermix (Bio-Rad, United States), 0.5 μL of each primer (10 μM), and 15 ng of DNA template. For all genes analyzed, the thermal cycling conditions were as follows: 95°C for 3 min, 45 cycles at 95°C for 15 s, annealing temperature (Tm) specific for each gene and primer pair for 30 s, and 72°C for 30 s. The fluorescence signal was read after each elongation step.

Amplicon specificity for each gene was verified by performing a melting curve starting at 55°C and increasing by 0.5°C until 95°C. Moreover, preliminary PCR was conducted to test the efficiency and the quality of primers (and amplicons), running gel electrophoresis of amplified DNA, and matching DNA bands with the expected fragment size.

Each assay was run in triplicate including the no template controls. Any possible qPCR inhibition was assessed by conducting an inhibition test using samples diluted from 10- to 100-fold, and no inhibition was observed. The quantitative PCR data were expressed as the ratio of ARG or intI1 gene copy number per 16S copy number to evaluate the relative proportion of each target gene in the bacterial community.

### Microbial Community Composition by Next-Generation Sequence

Aliquots of the DNA extracted (15 ng μL^–1^) from each replicate were used for NGS. The V3–V4 region of 16S rRNA genes was amplified with the Pro341F and Pro805R primers reported in Supplementary Table 3. These primers were selected in order to ensure the simultaneous identification of *Bacteria* and *Archaea* (Takahashi et al., 2014), both of which are involved in AD processes.

The size of the amplicons was checked with an agarose gel (1%), together with sample quality. The amplicons were sequenced with a MiSeq instrument (Illumina, Chesterford, United Kingdom) using the MiSeq Reagent Kit v3 for 600 cycles. The reads were imported and demultiplexed using QIIME 2 v2019.1^1^ and denoised with the DADA2 (Callahan et al., 2016) plugin. The primers were removed using the “*trim-left-f*” (forward) and “*trim-left-r*” (reverse) primer DADA2 functions. These functions remove the sequences from the beginning to a specific position. The exact length of the primers was 17 nucleotides for the forward and 21 nucleotides for reverse one. The amplicon sequencing variants obtained were compared to the 97% identical clustered Ribosomal Database Project (RDP release 11) using a naive Bayes classifier trained on the amplified region with 80% confidence. The principal coordinate analysis based on the Bray–Curtis distance and PERMANOVA as a statistical test was performed using the online tool Microbiome Analyst^2^ in order to evaluate the composition of the bacterial community during the AD process.

### Statistical Analysis

The sequenced data obtained were filtered with a minimum count of four and prevalence in samples of 20%. All samples were rarefied to an even sequence depth based on the sample having the lowest sequencing depth. The principal coordinate analysis based on the Bray–Curtis distance was performed using Past 3.25 (Hammer et al., 2001) in order to evaluate the composition of the bacterial community in different conditions. The comparison between conditions was performed using STAMP 2.1.3 (Parks et al., 2014) with the two-way ANOVA statistical test and Tukey-Kramer *post hoc* test. The *t*-test between two groups was performed using MS Excel 2013.

## Results and Discussion

### Biogas Production and Microbial Abundance

**FIGURE 1:**
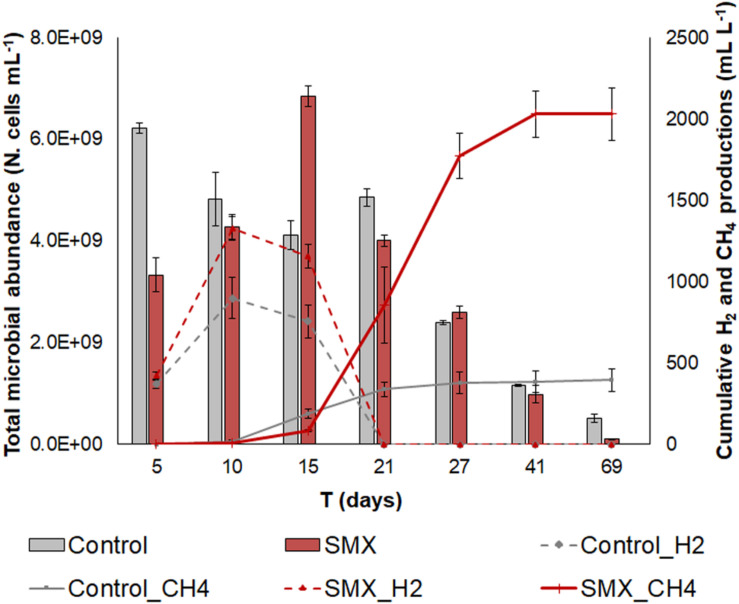
Total microbial abundance (N. cells mL^– 1^) in SMX-spiked (SMX) and control batches (histograms); cumulative biogas (CH_4_ and H_2_) production (lines) over the experimental time.

H_2_ and CH_4_ were produced in both SMX and control batches. In both cases, the production of H_2_ started at day 3 and continued until days 10–15, when the CH_4_ production also started (Figure 1).

However, a lower production (*p* < 0.01) of hydrogen as well as of methane was observed in the controls than in the SMX batches (Figure 1). In particular, the cumulative CH_4_ production was about five times higher in the SMX condition than the control one (2030.6 ± 143.3 mL L^–1^ vs. 386.8 ± 65.4 mL L^–1^). Furthermore, the maximum methane concentration was observed (71% from day 27) in SMX, and in control it never exceeded 31%. The highest H_2_ productions of 1239.6 ± 72.3 mL L^–1^ for SMX and 899.6 ± 83.3 mL L^–1^ for control, respectively, were reached at day 10. Subsequently, the cumulative H_2_ values decreased, and they were never found from day 21 (Figure 1). Contrary to these results, other authors (Cetecioglu et al., 2012) reported that biogas (CH_4_ and H_2_) production was not affected by adding SMX (Cetecioglu et al., 2012) at concentrations of 1 and 10 mg L^–1^ in an anaerobic system. However, an increase in hydrogen production was also observed in the presence of the antimicrobial triclocarban (Wang et al., 2019). To the best of our knowledge, the results of the experiment reported here are the first finding an increase in methane production when SMX was present at a 5 mg L^–1^. The presence of the SMX antibiotic favored the production of H_2_, which, in turn, presumably favored the production of CH_4_. The stimulation of H_2_ production due to bioaugmentation with a bacterial pool of hydrogen producers also led to an increase in methane production in another previous work (Ferraro et al., 2018). High hydrogen production is an indicator of the imbalance between the hydrolysis/acidogenesis and methanogenesis phases during the AD process. The results of this work can be ascribed to a high substrate/microorganisms ratio of the inoculum, which presumably promoted an enrichment of the hydrogen-producing bacteria, as is also found in another work (Yuan et al., 2019).

The initial microbial cell abundance (day 5) was higher in the control than in the SMX condition, showing an initial negative effect by the antibiotic on the microbial community (Figure 1). However, an increasing trend was observed in the SMX batches with a peak at day 15 when methane production started. At the end of the experiment, no differences in cell numbers were observed between the SMX and control conditions.

**FIGURE 2:**
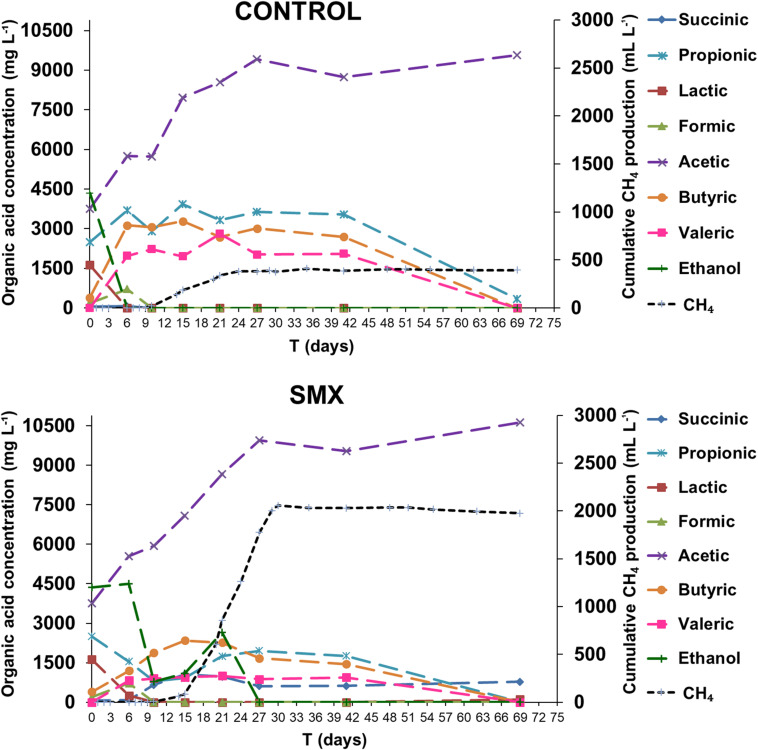
Organic acid concentrations and ethanol during the AD process (the standard deviations range from 7.6 to 13.4%).

The process intermediate compositions, which include VFAs, succinic and lactic acids, and ethanol, are reported in Figure 2. In the ingestate, high concentrations of acetic, propionic, and lactic acid (3.7 g L^–1^, 2.5 g L^–1^, and 1.6 g L^–1^, respectively) and ethanol (4.3 g L^–1^) were measured. The pH value (pH 6.4) at the start of the experiment was higher than the acids’ dissociation constant (≤4.86 at 25°C); this result suggests that the VFAs were mainly in their dissociated forms and, therefore, unable to permeate inside cells and inhibit the microbial activity (Wainaina et al., 2019). Although the high concentration of soluble metabolites suggests that the methanogenesis and acetogenesis started quickly, a high hydrogen production was also observed. Hydrogen production occurred immediately in both experimental conditions, and the acetic, butyric, formic, succinic, and valeric acids were detected, indicating a mixed acid fermentation. Moreover, a simultaneous consumption of lactic acid and ethanol for both experimental conditions was detected, indicating an early acetogenic pathway. The oxidation of lactate and ethanol is inhibited when hydrogen partial pressure is approaching 1 ATM, as evaluated by thermodynamic calculations (Harper and Pohland, 1986). The highest calculated values of H_2_ partial pressure were 0.47 and 0.70 ATM in the control and SMX conditions, respectively (day 6). During hydrogen production, the pH dropped (day 3) to values of 4.8 and 5.1 in control and SMX, respectively. In SMX, it increased to 6.3 (day 21) and gradually decreased to 5.4 at the end of experiment. In the control condition, the highest pH value was 6.0 (day 15), but it quickly decreased to 5.5 (day 21). During methane production, a high production of ethanol (2.6 g L^–1^) and a further acetic acid increase were observed from 15 to 21 days in the SMX condition. Subsequently, the ethanol was promptly consumed, and the methane production reached its maximum value; in this case, a late fermentative acetate–ethanol pathway followed by an ethanol acetogenic phase can be hypothesized. Another relevant difference between the two experimental conditions was the total VFA concentration over time: In control batches, a value of 17 g L^–1^ was reached at day 15, decreasing to 9.92 g L^–1^ at the end of AD (day 69); in the SMX condition, a lower value than 15.0 g L^–1^ was measured over the AD process. The latter results are in accordance with a recent experimental study on codigestion of food waste and pig manure, which reports that the threshold inhibition concentration of VFAs ranged from 16.5 to 18.0 g L^–1^ (Jiang et al., 2018). However, in the present experiment, hydrogenotrophic metabolism was the active pathway for methane production, particularly for SMX. In this condition, the hydrogen production resulted mainly from the acidogenic phase as well as from lactic acid and ethanol acetogenesis. These results are in line with the fact that methane production from acetic acid is inhibited by H_2_ partial pressure above 10^–4^ ATM and that propionic acid oxidation to acetate is favorable only at H_2_ partial pressure below 10^–4^ ATM. Moreover, butyric acid oxidation is favorable at H_2_ partial pressures of 10^–3^ ATM or below. For these reasons, the production of methane from H_2_/CO_2_ is favored compared to acetate cleavage when H_2_ partial pressure is above 10^–4^ ATM. In the present work, the threshold H_2_ partial pressure of 10^–4^ ATM was reached at day 41, when an oxidation of VFAs and a further accumulation of acetic acid were observed, suggesting that the acetogenesis phase was coupled with homoacetogenesis, as demonstrated by the absence of methane and hydrogen production.

### Microbial Community Structure

The FISH analysis of the ingestate used to start the experiment showed a predominance of *Bacteria* (92.2%) corresponding to 1.74 10^9^ cells mL^–1^ and a presence of *Archaea* (0.6%) corresponding to 1.13 10^7^ cells mL^–1^. The NGS sequencing of the ingestate supported this FISH analysis, showing similar initial percentages of 99% of *Bacteria* and 0.8% of *Archaea*, respectively. Among *Bacteria*, the main groups found were *Clostridia* (38%) *Bacteroidia* (26%), and *Bacilli* (14%) followed by Gamma-*Proteobacteria* (11%) and *Erysipelotrichia* (5%). Minor groups were *Actinobacteria* (4%), *Synergista* (1%), and *Verrucomicrobiae* (1%).

**FIGURE 3:**
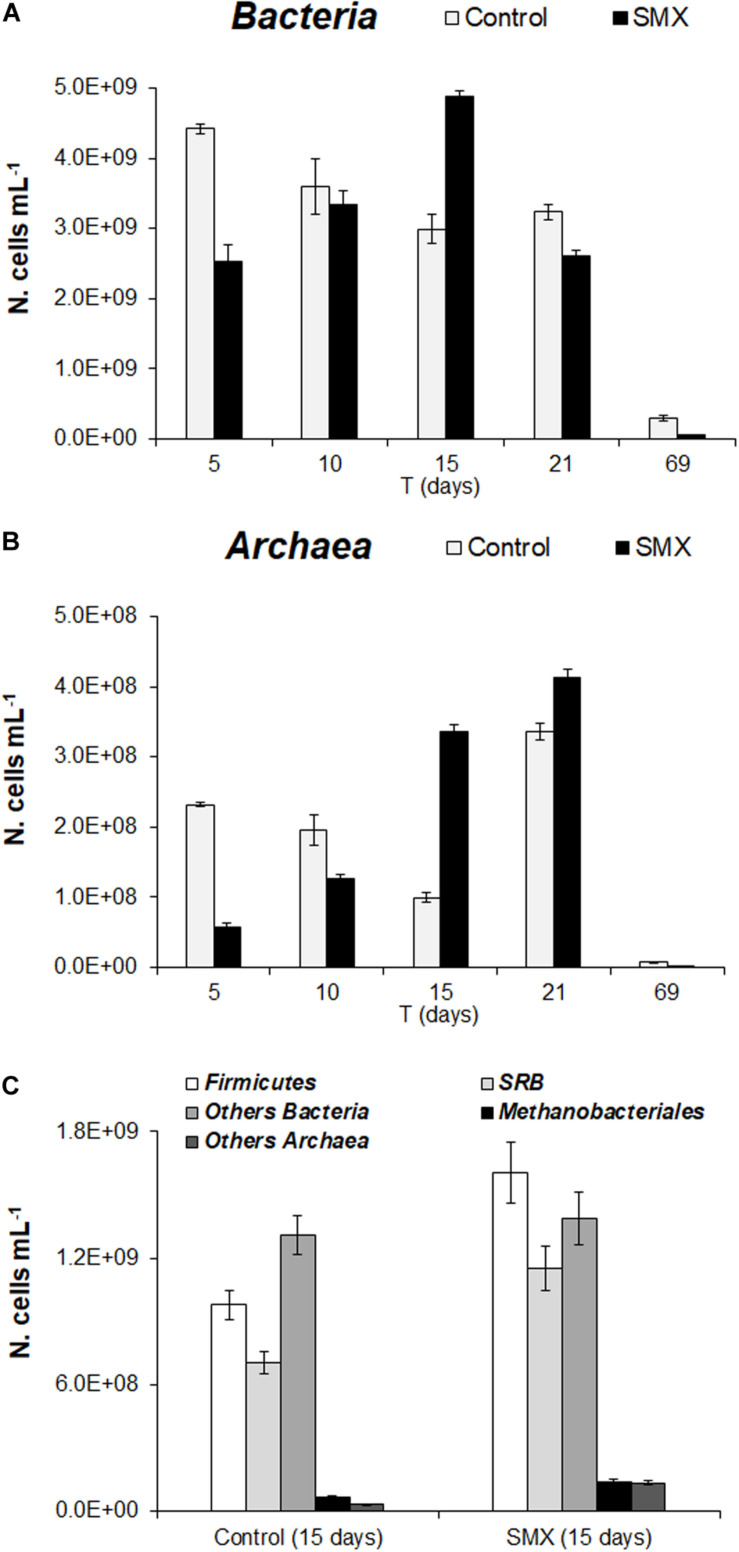
**(A)** N. cells mL^– 1^ of *Bacteria* and **(B)** *Archaea* in control and SMX-spiked (SMX) batches over the experimental time evaluated by FISH. **(C)** N. cell mL^– 1^ of *Firmicutes*, SRB, and *Methanobacteriales* detected at day 15.

In the batch experiment, the FISH analysis showed an initial detrimental antibiotic effect (*t*-test, *p* < 0.01) both for *Bacteria* and *Archaea*, as shown by the significantly lower number of cells (N. cells mL^–1^) in SMX than in control at 5 days (Figures 3A,B). These results are in line with those of the total microbial abundance and show how SMX, at the initial concentration used in this experiment, negatively affected some microbial populations. However, this effect was transient, and an increase in *Bacteria* was observable until day 15 (at the end of H_2_ production) and subsequently in *Archaea* (Figures 3A,B). In particular, an increase in *Archaea* abundance was detected from days 15 and 21 for SMX and control, respectively (Figure 3B) conferring a more balanced structure to the microbial community in line with the highest rate of methane production and hydrogen consumption. The FISH analysis made it possible to visualize and quantify at day 15, a significant increase in the abundance of *Firmicutes* and in the functional group of SRB in the SMX condition compared to control (Figure 3C). *Firmicutes* is one of the most abundant populations found to compose the bacterial populations of the AD community, and their increase can be related to an improvement in the process performance (Chen et al., 2016).

Sulfate-reducing bacteria form a functional group able to use H_2_ for producing H_2_S, competing with methanogenic bacteria in the metabolic pathway of H_2_/CO_2_; on the contrary, in this work, SRB activity presumably helped to consume H_2_, contrasting the lowering of pH and, therefore, promoting the performance of the process.

**FIGURE 4:**
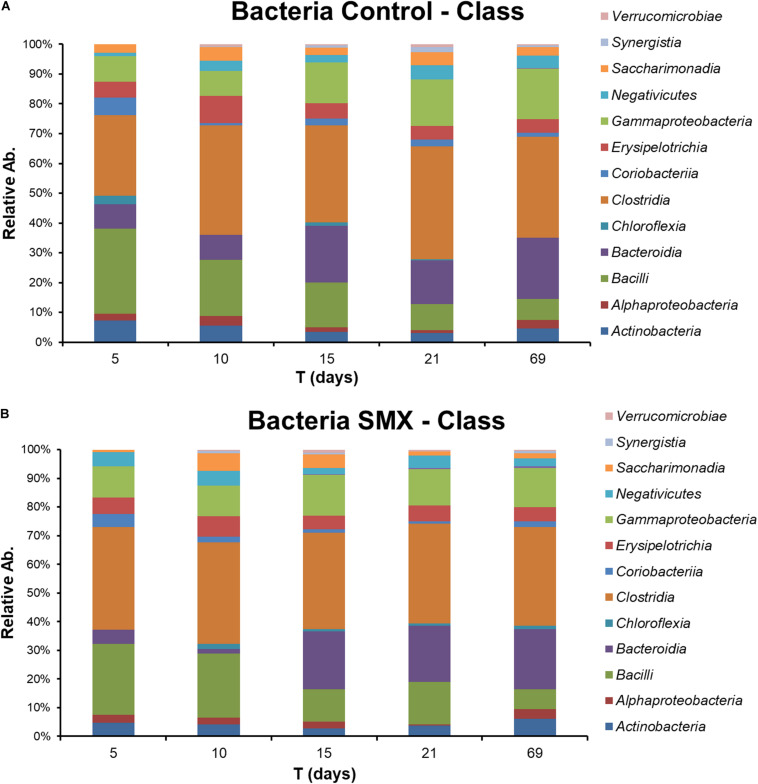
Relative abundance of Bacteria at different experimental times evaluated by NGS in Control **(A)** and **(B)** SMX batches.

**FIGURE 5:**
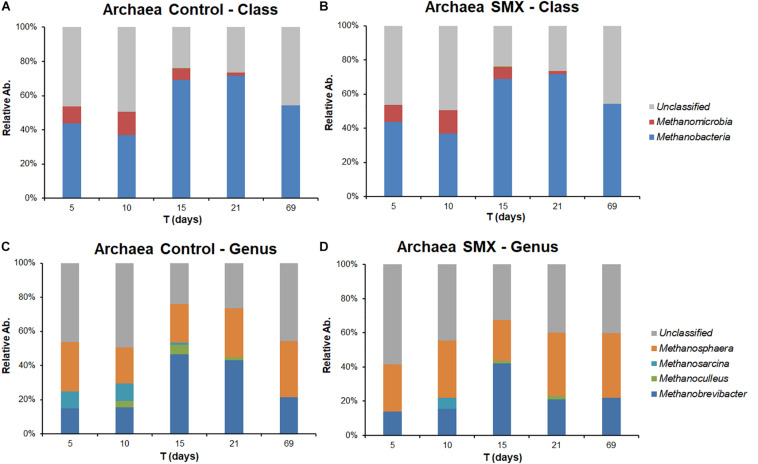
Relative abundance of *Archaea* in control and SMX conditions evaluated by NGS at class **(A,B)** and genus level **(C,D)**, respectively.

The NGS results (Figures 4A,B, 5A–D) provide a deeper insight into the microbial community and its dynamic during the AD process and made it possible to show its shifts under the experimental conditions at different phylogenetic levels. The Chao index calculated for estimating the number of species in a community (Jiwon et al., 2017) was significantly (*t*-test, *p* < 0.01) different between the ingestate and the control and SMX conditions (Table 2). A higher value (Two-way ANOVA, *p* < 0.01) of this index was observed in the control than in the SMX over the experimental time. These results show a direct effect of the antibiotic on the microbial community.

**TABLE 2:** Diversity indices in the ingestate and during the anaerobic digestion.

	Diversity index		
	Chao1	Shannon	Evenness
Ingestate	52	7.86	0.93
Control 5 days	82	7.59	0.95
SMX 5 days	77	7.26	0.94
Control 10 days	95	7.62	0.95
SMX 10 days	85	7.29	0.94
Control 15 days	118	8.02	0.95
SMX 15 days	87	7.71	0.95
Control 21 days	89	7.53	0.95
SMX 21 days	79	7.17	0.94
Control 69 days	89	7.49	0.94
SMX 69 days	75	7.09	0.94

**FIGURE 6:**
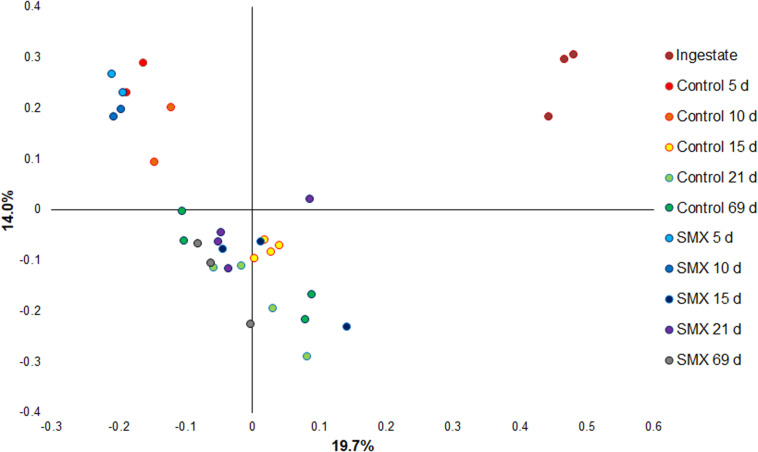
Principal coordinate analysis based on Bray–Curtis (Permanova *p* < 0.001) for evaluating the difference in composition of the bacterial community under the different conditions.

Moreover, the Bray–Curtis principal coordinate analysis shows significant differences (Permanova, *p* < 0.001) between the ingestate, the first experimental phase of H_2_ production (days 5–10), and the second phase of CH_4_ evolution (days 15–69), demonstrating how the microbial community changed during the AD process (Figure 6).

Significant differences were found inside some archaeal and bacterial groups between the control and SMX batches at day 5. In particular, significantly lower percentages (*p* < 0.01) of *Methanomicrobia* (10% control vs. 0% SMX), *Bacilli* (28% control vs. 25% SMX) and *Bacteroidia* (8% control vs. 5% SMX) were found in the SMX batches.

The archaeal fraction was mainly composed of two classes: *Methanomicrobia* and *Methanobacteria*. At genus level, two genera predominated, *Methanobrevibacter* (26%) and *Methanosphaera* (17%), which are known to thrive in a range of pH values between 5.0 and 7.5 (Madigan et al., 2014) in line with the values recorded in this experiment (Figure 5A–D). In particular, *Methanobrevibacter* is acid-tolerant and can grow from pH 5.0 to 7.5 (Savant et al., 2002). The methane production involves mainly acetotrophic (e.g., *Methanosarcinales*) and hydrogenotrophic archaeal groups, the former producing methane using acetic acid and the latter using H_2_/CO_2_ (Castellano-Hinojosa et al., 2018); the results of the present work show that, in the control condition, there was a higher percentage (*p* < 0.01) of *Methanomicrobia* than in the SMX one (Figure 5). Inside the class *Methanomicrobia*, the genus *Methanosarcina*, able to use acetic acid for producing methane, was detected in the control condition at days 5, 10, and 15 (9.3, 9.9, and 1.3% respectively), and in the SMX one only at day 10 (6.0%). The overall microbial community characterization results compared with the VFA production trend (Figure 2) clearly indicate that hydrogenotrophic metabolism was the active pathway for methane production, especially for the SMX condition. The acetoclastic pathway was presumably inactive as can be deduced from the accumulation of acetic acid as well as the absence or low amount of *Methanosarcina* in SMX.

### ARGs and SMX Analyses

The relative abundance (ARG/16S) of *sul1*, *sul2*, and *intI1* genes in the ingestate and in the batch experiment are reported in Table 3. The most abundant genes detected in the ingestate were *sul1* and the proxy *intI1*, and the ARG *sul2* was detected in a lower concentration. The overall abundance of ARGs decreased in the digestate anaerobic process in both SMX and control conditions with significantly lower values (*p* < 0.01) at the end of the experiment. However, in the control the residual concentration of SMX (ca. 0.30 mg L^–1^) found in the ingestate still persisted (Table 3).

**TABLE 3:** Relative abundance (ARG 16S^–1^ ± standard error) of *sul1*, *sul2*, and *intI1* and quantification of SMX (mg L^–1^ ± standard error) in the ingestate (0 days) and batch experiments at 15 and 69 days.

	0 days	15 days		69 days	
	Ingestate	Control	SMX	Control	SMX
*sul1* (ARG 16S^–1^)	5.5 ± 0.5	0.0010 ± 0.0005	0.0004 ± 0.0003	0.14 ± 0.09	0.077 ± 0.05
*intI1* (ARG 16S^–1^)	0.35 ± 0.25	0.06 ± 0.04	0.4 ± 0.2	0.3 ± 0.2	0.04 ± 0.02
*sul2* (ARG 16S^–1^)	0.0010 ± 0.0008	0	0	0	0
SMX (mg L^–1^)	0.3 ± 0.1	0.3 ± 0.2	3.7 ± 0.1	0.3400 ± 0.002	0.9 ± 0.2

The antibiotic concentration decreased over time in the SMX-spiked batches and in line with the increase in microbial abundance; at the end of the experiment, about 80% was degraded. This result shows that SMX can also be degraded in anaerobic conditions; SMX biodegradation has been found in previous works in effluents (Patrolecco et al., 2018) and digestate-treated soil in aerobic conditions (Rauseo et al., 2019). Recent works (Wang et al., 2016; Gurmessa et al., 2020) report SMX to be removed mainly by biodegradation and that adsorption processes were negligible. Moreover, in AD experiments, in which the substrate was pig manure, SMX was removed with variable elimination rates from 0 to 100%, depending on specific process parameters, such as temperature and hydraulic retention time (Gurmessa et al., 2020).

## Conclusion

The results show a selective effect of SMX on the microbial community, which not only did not hamper AD, but also stimulated some microbial populations involved in biogas production and in the antibiotic degradation.

The AD process promoted an overall lowering of the ARG load inside the microbial community in both control and SMX conditions. Interestingly, the decrease in the relative abundance of ARGs was not directly related to the reduction in antibiotic concentration. This supports the hypothesis that whatever the initial antibiotic concentration, the AD process seems to discourage ARG maintenance among the microbial community, owing presumably to its fitness costs. Further studies are in progress for evaluating in real biogas plants if the AD process, with manure as feedstock, is able to decrease the antibiotic residues and ARGs. The latter aspect is particularly important because AD might lower the risk of ARG spread and make digestate more suitable than manure for replacing chemical fertilizers.

## Data Availability Statement

The datasets generated for this study are available at: https://www.ebi.ac.uk/ena/browser/view/PRJEB37044.

## Author Contributions

All authors listed have made a substantial, direct and intellectual contribution to the work, and approved it for publication.

## Conflict of Interest

The authors declare that the research was conducted in the absence of any commercial or financial relationships that could be construed as a potential conflict of interest.

## Footnotes

1 https://qiime2.org

2 http://www.microbiomeanalyst.ca
