# Supplementary material for: Effects of Sulfamethoxazole on the Microbial Community Dynamics During the Anaerobic Digestion Process
Source: Front Microbiol. 2020 Sep 16;11:537783. doi: 10.3389/fmicb.2020.537783 (PMC7525162; doi:10.3389/fmicb.2020.537783)

Supplementary Material

Supplementary Figure 1. Micrograph of the overall microbial community of the ingestate obtained by DAPI staining

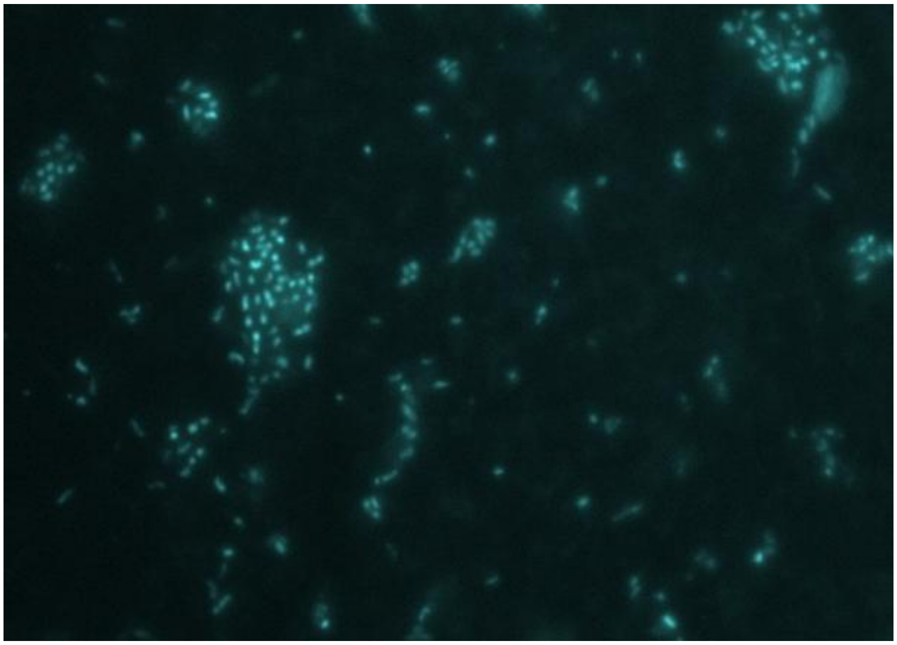

Supplement: Supplementary file 1 [file Image_1.PDF]
