# Supplementary material for: Effects of Sulfamethoxazole on the Microbial Community Dynamics During the Anaerobic Digestion Process
Source: Front Microbiol. 2020 Sep 16;11:537783. doi: 10.3389/fmicb.2020.537783 (PMC7525162; doi:10.3389/fmicb.2020.537783)
Supplement: Supplementary file 2 [file Table_1.DOCX]

Supplementary Material

**Supplementary Table 1.** Quantification of metals in the ingestate used for the batch experiment

| Metal | Li | Be | B | Na | Mg | Al | K | Ca | V | Cr | Mn |
| --- | --- | --- | --- | --- | --- | --- | --- | --- | --- | --- | --- |
| mg kg^-1^ | 0.9 | <0.05 | <0.05 | 4307 | 1608 | 1317 | 19807 | 12224 | 1.0 | 1.6 | 152 |
| Metal | **Fe** | **Co** | **Ni** | **Cu** | **Zn** | **Ga** | **As** | **Se** | **Rb** | **Sr** | **Mo** |
| mg kg^-1^ | 563 | <0.05 | 2.8 | 20 | 128 | 3.2 | <0.05 | <0.05 | 73.4 | 32.8 | 2.9 |
| Metal | **Ag** | **Cd** | **Sb** | **Te** | **Cs** | **Ba** | **Hg** | **Tl** | **Pb** | **Bi** | **U** |
| mg kg^-1^ | 2.2 | <0.05 | <0.05 | <0.05 | <0.05 | 28.9 | <0.05 | <0.05 | <0.05 | <0.05 | <0.05 |
