# Supplementary material for: Effects of Sulfamethoxazole on the Microbial Community Dynamics During the Anaerobic Digestion Process
Source: Front Microbiol. 2020 Sep 16;11:537783. doi: 10.3389/fmicb.2020.537783 (PMC7525162; doi:10.3389/fmicb.2020.537783)
Supplement: Supplementary file 3 [file Table_2.DOCX]

Supplementary Material

**Supplementary Table 2.** List of all primers used for qPCR

| Target gene | Primer set | Sequence (5’ - 3’) | Amplicon length (bp) | Annealing temperature (°C) | Reference |
| --- | --- | --- | --- | --- | --- |
| *sul1* | Sul1 fw | CGCACCGGAAACATCGCTGCAC | 161 | 60 | (Vila-Costa et al., 2017) |
|  | Sul1 rv | TGAAGTTCCGCCGCAAGGCTCG |  | 60 |  |
| *sul2* | Sul2 fw | GCGCTCAAGGCAGATGGCATT | 284 | 57 | (Kerrn et al., 2001) |
|  | Sul2 rv | GCGTTTGATACCGGCACCCGT |  | 57 |  |
| *intI1* | Int 1f2 | TCGTGCGTCGCCATACA | 66 | 52 | (Byrne-Bailey et al., 2011) |
|  | Int 1r2 | GCTTGTTCTACGGCCGTTTGA |  | 52 |  |
| *16S rRNA* | 16s-fw | CGGTGAATACGTTCYCGG | 123 | 57 | (Mckinney et al., 2018) |
|  | 16s-rv | TACCTTGTTACGACTT |  | 53 |  |

**References**

Byrne-Bailey, K. G., Gaze, W. H., Zhang, L., Kay, P., Boxall, A., Hawkey, P. M., et al. (2011). Integron prevalence and diversity in manured soil. *Appl. Environ. Microbiol.* doi:10.1128/AEM.01425-10.

Kerrn, M. B., Klemmensen, T., Espersen, F., and Frimodt-Møller, N. (2001). Control of resistance to sulphonamides. *Lancet*. doi:10.1016/s0140-6736(01)05915-3.

Mckinney, C. W., Dungan, R. S., Moore, A., and Leytem, A. B. (2018). Occurrence and abundance of antibiotic resistance genes in agricultural soil receiving dairy manure. 1–10. doi:10.1093/femsec/fiy010.

Vila-Costa, M., Gioia, R., Aceña, J., Pérez, S., Casamayor, E. O., and Dachs, J. (2017). Degradation of sulfonamides as a microbial resistance mechanism. *Water Res.* doi:10.1016/j.watres.2017.03.007.
