# Supplementary material for: Effects of Sulfamethoxazole on the Microbial Community Dynamics During the Anaerobic Digestion Process
Source: Front Microbiol. 2020 Sep 16;11:537783. doi: 10.3389/fmicb.2020.537783 (PMC7525162; doi:10.3389/fmicb.2020.537783)
Supplement: Supplementary file 4 [file Table_3.DOCX]

Supplementary Material

**Supplementary Table 3.** List of primers used for metagenomic analysis

| **Primer name** | **Oligonucleotide sequence (5’–3’)** | **Reference** |
| --- | --- | --- |
| Pro341F | AATGATACGGCGACCACCGAGATCTACACTCTTTCCCTACACGACGCTCTTCCGATCTCCTACGGGAGGCAGCAG**CCTACGGGNBGCASCAG** | (Takai and Horikoshi, 2000) |
| Pro805R | CAAGCAGAAGACGGCATACGAGATNNNNNNGTGACTGGAGTTCAGACGTGTGCTCTTCCGATCT**GACTACNVGGGTATCTAATCC** | (Herlemann et al., 2011) |

**References**

Herlemann, D. P. R., Labrenz, M., Jürgens, K., Bertilsson, S., Waniek, J. J., and Andersson, A. F. (2011). Transitions in bacterial communities along the 2000 km salinity gradient of the Baltic Sea. *ISME J.* doi:10.1038/ismej.2011.41.

Takai, K., and Horikoshi, K. (2000). Rapid detection and quantification of members of the archaeal community by quantitative PCR using fluorogenic probes. *Appl. Environ. Microbiol.* doi:10.1128/AEM.66.11.5066-5072.2000.
